# Unintended consequences of implementing non-pharmaceutical interventions for the COVID-19 response in Africa: experiences from DRC, Nigeria, Senegal, and Uganda

**DOI:** 10.1186/s12992-023-00937-6

**Published:** 2023-06-06

**Authors:** Issakha Diallo, Rawlance Ndejjo, Mamadou Makhtar Mbacké Leye, Landry Egbende, Andrew Tusubira, Eniola A. Bamgboye, Manel Fall, Noel Namuhani, Marc Bosonkie, Mobolaji M. Salawu, Youssoupha Ndiaye, Steven Ndugwa Kabwama, Ndeye Mareme Sougou, Segun Bello, Omar Bassoum, Ziyada Babirye, Rotimi Felix Afolabi, Thiané Gueye, Susan Kizito, Ayo S. Adebowale, Magbagbeola David Dairo, William Sambisa, Suzanne N. Kiwanuka, Olufunmilayo I. Fawole, Mala Ali Mapatano, Rhoda K. Wanyenze, Ibrahima Seck

**Affiliations:** 1 Public Health Department, Faculty of Health Sciences, University Amadou Hampaté Ba, Dakar, Senegal; 2 Department of Disease Control and Environmental Health, School of Public Health, College of Health Sciences, Makerere University, Kampala, Uganda; 3 Preventive Medicine and Public Health Department within the Faculty of Medicine, Pharmacy and Dentistry, University Cheikh Anta Diop of Dakar, Dakar, Senegal; 4 Kinshasa School of Public Health, Kinshasa, Democratic Republic of the Congo; 5 Department of Community Health and Behavioural Sciences, Makerere University School of Public Health, Kampala, Uganda; 6 Department of Epidemiology and Medical Statistics, Faculty of Public Health, College of Medicine, University of Ibadan, Ibadan, Nigeria; 7 Epidemiology Department of Pasteur Institute of Dakar, Dakar, Senegal; 8 Department of Health Policy, Planning and Management, School of Public Health, College of Health Sciences, Makerere University, Kampala, Uganda; 9 Health Economics Unit of the Ministry of Health and Social Action, Dakar, Senegal; 10 Bill and Melinda Gates Foundation, Seattle, USA

**Keywords:** COVID-19, Unintended Consequences, NPI, Stringency, DRC, Nigeria, Senegal, Uganda

## Abstract

**Introduction:**

The coronavirus (COVID 19) pandemic is one of the most terrifying disasters of the twenty-first century. The non-pharmaceutical interventions (NPIs) implemented to control the spread of the disease had numerous positive consequences. However, there were also unintended consequences—positively or negatively related to the nature of the interventions, the target, the level and duration of implementation. This article describes the unintended economic, Psychosocial and environmental consequences of NPIs in four African countries.

**Methods:**

We conducted a mixed-methods study in the Democratic Republic of Congo (DRC), Nigeria, Senegal and Uganda. A comprehensive conceptual framework, supported by a clear theory of change was adopted to encompass both systemic and non-systemic interventions. The data collection approaches included: (i) review of literature; (ii) analysis of secondary data for selected indicators; and (ii) key informant interviews with policy makers, civil society, local leaders, and law enforcement staff. The results were synthesized around thematic areas.

**Results:**

Over the first six to nine months of the pandemic, NPIs especially lockdowns, travel restrictions, curfews, school closures, and prohibition of mass gathering resulted into both positive and negative unintended consequences cutting across economic, psychological, and environmental platforms. DRC, Nigeria, and Uganda observed reduced crime rates and road traffic accidents, while Uganda also reported reduced air pollution. In addition, hygiene practices have improved through health promotion measures that have been promoted for the response to the pandemic. All countries experienced economic slowdown, job losses heavily impacting women and poor households, increased sexual and gender-based violence, teenage pregnancies, and early marriages, increased poor mental health conditions, increased waste generation with poor disposal, among others.

**Conclusion:**

Despite achieving pandemic control, the stringent NPIs had several negative and few positive unintended consequences. Governments need to balance the negative and positive consequences of NPIs by anticipating and instituting measures that will support and protect vulnerable groups especially the poor, the elderly, women, and children. Noticeable efforts, including measures to avoid forced into marriage, increasing inequities, economic support to urban poor; those living with disabilities, migrant workers, and refugees, had been conducted to mitigate the negative effects of the NIPs.

## Introduction

Epidemics have the terrible peculiarity of spreading rapidly, causing very high rates of morbidity, mortality and even disability in certain cases. The coronavirus SARS-CoV-2 also referred to as COVID-19, an RNA virus first discovered on November 16, 2019, caused a global pandemic in less than three months [1]. As of 31^st^ December 2020, the global cumulative incidence reached 197,195,568 reported cases and 4,210,201 associated deaths with a case fatality ratio (CFR) of 2.1%. This rate was 2.4% (CFR) in Africa which recorded 4,917,071 cases or 2,5% [2] of the total cases and 117,304 associated deaths within the period for a population estimated at 17.2% of the world’s population. Nigeria, DRC, and Senegal were the first countries to record COVID-19 cases in sub-Saharan Africa in March of 2020 [3]. Across the African continent, the southern region had the highest burden of COVID-19 compared to other regions [4]. There was no clear difference in the number of cumulative confirmed cases of COVID-19 cases per million population between countries in the Eastern, Central, and Western regions of the continent, except for the United Republic of Tanzania, which stopped reporting COVID-19 cases to World Health Organization (WHO)Africa Regional Office on the 7^th^ of May, 2020 [5, 6]. However, considering the new daily confirmed cases per million, Africa was in its third wave in July of 2020, and the eastern region, particularly Uganda, had a high disease burden compared to countries in Central or West Africa. The four countries covered by this study, Nigeria, Uganda, the DRC, and Senegal, had a total of 358,640 cases and 6,757 deaths recorded as of the 31^st^ of December 2020.

The COVID-19 pandemic is an excellent illustration of the occurrence of a rapid spread of communicable disease, facilitated by the enormity and diversity of means of transport with people moving frequently all over the world. Given its high contagiousness and the lack of effective drugs or vaccinations in the early phase, governments relied on non-pharmaceutical interventions (NPIs) to slow the spread of the virus. While these measures may have been effective, they also led to considerable unintended consequences. These interventions were, in many countries, legally, and ethically in conformity with the public health laws of communities/nations and the rights of individuals, as well as national laws and international treaties. In countries where NPIs were not aligned with existing public health laws, amendments were made, or new regulations were enacted (e.g. in Uganda) to enable their implementation. As of the 25^th^ of February 2020, zero cases had been reported in Africa [7]. The application of NPIs began in Africa in March of 2020. Rwanda was the first to implement lockdowns on 21^st^ of March, followed by Nigeria on the 27^th^ of March. Subsequently, most of the countries introduced total or partial containment NPIs [8].

African countries were already suffering from the high burden of infectious diseases (both endemic and epidemic), increase in non-communicable diseases, and weak health systems. Unintended consequences were observed in the health, socio-economic and environmental sectors, exacerbated by poverty, fragile health systems and poor governance. This paper presents psychological, socio-economic, and environmental unintended consequences following the implementation of NPIs in the Democratic Republic of the Congo (DRC), Nigeria, Senegal, and Uganda.

### Conceptual framework & the study boundaries

The implementation of NPIs, like hand washing, wearing face coverings or physical distancing, was among the most effective methods of controlling the COVID-19 pandemic as recommended by WHO. NPIs transcend the limits of the health system to a transversal, multisectoral approach to health issues, integrating all the components of health protection [9] with a focus on preventive and promotional health measures. These interventions aimed at delaying the onset of an epidemic or pandemic and the timing of the outbreak peaks by reducing the number of people infected by the disease.

Classically, the evaluation of NPIs, like pharmaceutical interventions, seeks to justify the added value in improving the health of populations by these interventions. Thus, the majority of evaluations of NPIs implemented in the context of communicable diseases focus on their expected effects by examining the factors mitigating transmission. Despite their undeniable preventive benefits, NPIs have unexpected positive and negative externalities. These effects vary depending on the nature of the intervention, the sectors most affected, the rigor with which the intervention is applied, the level and scope, the duration and the opportunities and constraints associated with its implementation. Complex relationships emerge through and between consequences; expected, unexpected as summarized by Bloom Rosen’s conceptual framework [10]. Consequences can be grouped into two categories (i.e. anticipated and unanticipated). The categories were then grouped into direct and indirect outcomes. The effects of interventions can thus be anticipated and desired—these are not addressed in this study, which focuses on the unexpected effects, some of which are arbitrations between those that we anticipated but that we did not want (negative effects), serendipities, and desirable and unexpected and unwanted because they are negative. This study focuses on unintended consequences, excluding those arising from school closures, which are discussed elsewhere.

### Methodology

This was a multi-country cross-sectional study conducted from March 2020 to 31^st^ December 2020. in DRC, Nigeria, Senegal, and Uganda. These countries were selected because of their variability in the response to COVID-19 and in the resulting outcomes, their experience in managing previous outbreaks of global scope. Other selection criteria included the strong partnerships between the academic institutions and the ministries of health, which facilitated access to data and translated results into action, and the representation of Anglophone and Francophone communities to strengthen South-South collaboration.

#### Study design and data collection

The study used a mixed methods approach with: 1) a literature review, using reports, journal publications, ministerial statements, press articles and blogs on the unintended consequences of COVID-19 NPIs in the four countries; 2) analysis of secondary data on selected unintended outcomes; and 3) qualitative interviews with 25 to 30 key informants in each country. The key informants included policy makers from different ministries, civil society representatives, local leaders and law enforcement officials, to obtain a deeper understanding of the implementation of COVID-19 NPIs and their consequences. The interviews in all countries were conducted face-to-face with the interviewees after having obtained a well-scheduled meeting beforehand.

#### Data analysis

The triangulation of the results was carried out using different methodologies according to the dimensions of the different analysis frameworks. NVivo software, a qualitative analysis software, was used to identify different themes and nodes for triangulation. This software provides a structure / framework allowing coding of documents, videos, tweets etc. and speeds up analysis as well as easy sharing of documents. Finally, based on the analytical framework, the unintended consequences of NPIs were grouped into two categories: anticipated and unforeseen; positive and negative consequences. The categories were further classified into direct and indirect outcomes.

### Ethical considerations

The key informants provided voluntary informed verbal consent and anonymity, confidentiality observed in handling and communication of results. The study protocol was approved by the national ethics committees in each of the four countries before commencement of data collection in DRC, Nigeria, Senegal and Uganda.

## Results

### Overview of the pandemic and NPIs in the four countries

**Fig. 1:**
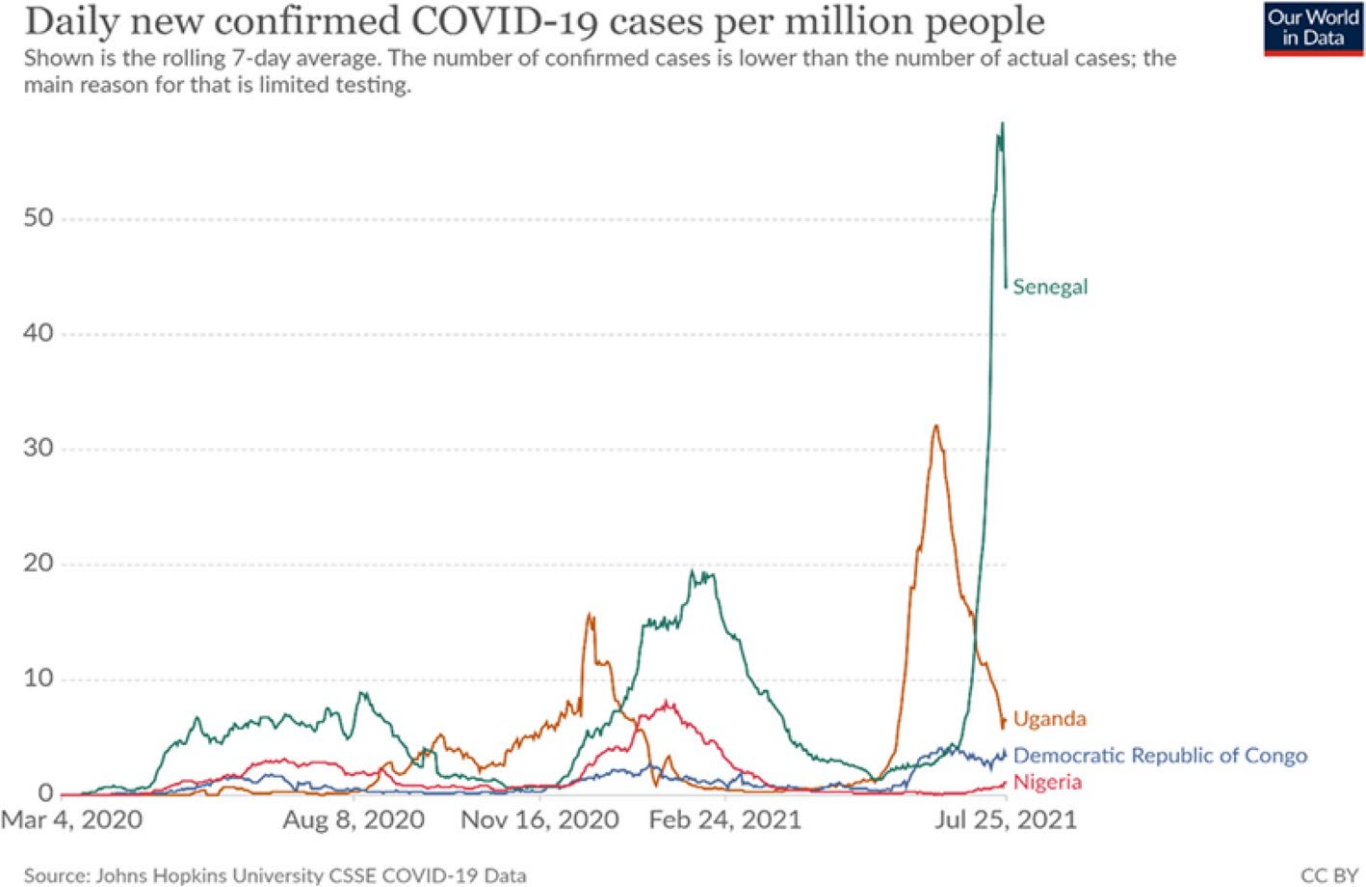
COVID-19 epidemic curve across DRC, Nigeria, Senegal, and Uganda

The literature review identified the cumulative number of COVID-19 cases and deaths in the four countries as of 31^st^ December 2020, was very high despite the implementation of NPIs. This was due to the emergence of the Delta variants and to laxity in the adherence to NPIs. Subsequent pandemic waves had higher peaks and occurred over a shorter period (Fig. 1).

### NPIs implemented in the four countries

**Fig. 2:**
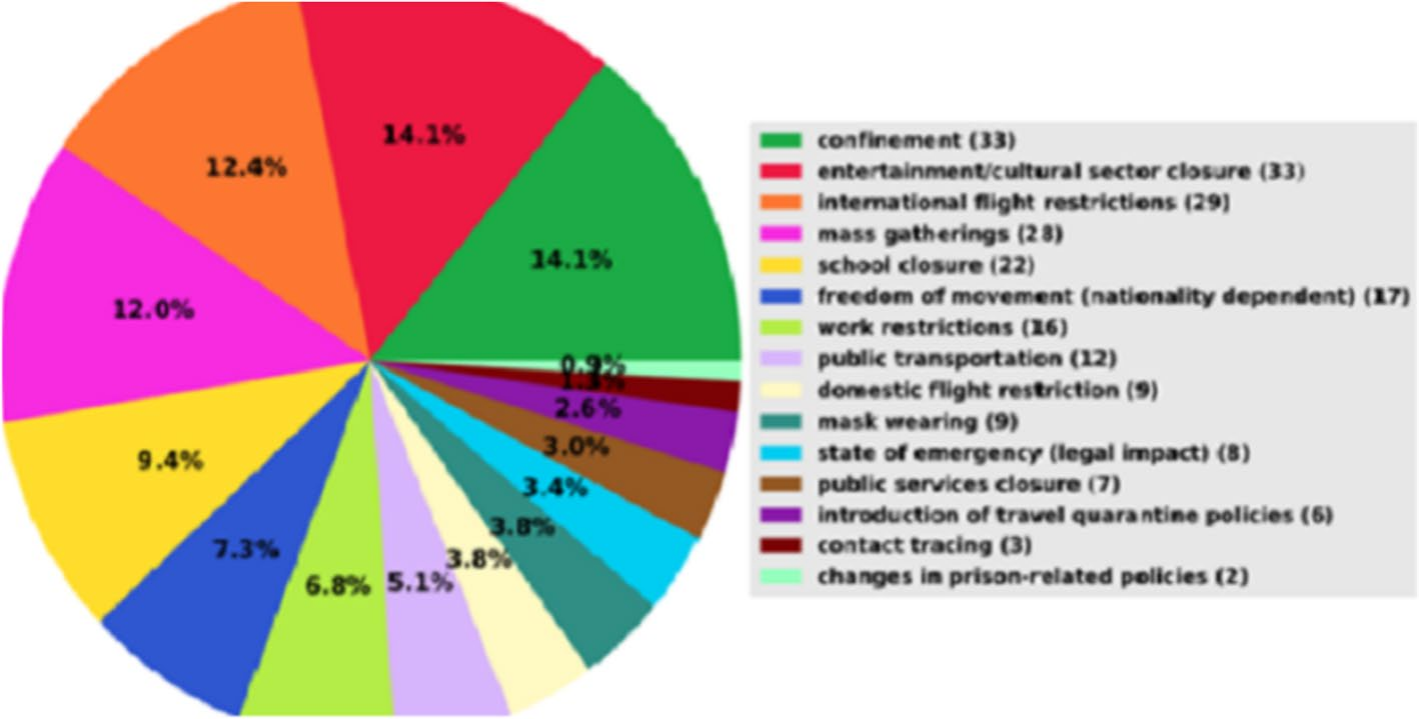
Distribution of NPIs in the four African countries; Nigeria, Uganda, DRC and Senegal

The study identified several NPIs as part of the COVID-19 response in Nigeria, Uganda, DRC, and Senegal, illustrated in Fig. 2 [11]. Based on the taxonomy of the Worldwide Non-pharmaceutical Interventions Tracker for COVID-19 (WNTRA) dataset for COVID-19, 16 categories of NPIs have been implemented worldwide. The four study countries implemented 15 of these 16 (94%) NPI categories. Figure 2 shows the distribution of the categories of NPIs implemented in the four countries by the 31^st^ December 2020. The NPIs included lockdowns (14.1%), closure of the entertainment/cultural sector (14.1%), international flight restrictions (12.4%) and mass gatherings (12.0%), amongst others.

These NPIs had unintended health, economic, socio-economic, psychosocial, environmental, and other consequences. Some of these consequences were positive and others negative. Several measures were taken to minimize the negative effects such as relief support to vulnerable groups, government economic measures and tax exemptions, building community resilience, strengthening data systems, psychosocial support, drawing on local resources, ensure the continuity of essential services, use of innovations, and technological solutions, among others. Most communities worked together to support each other to deal with the pandemic and its effects, notably to address their precarious financial situations. Social support in the form of family and community closeness and various coping mechanisms were also developed. In addition, to mitigate the economic and social impact of the coronavirus pandemic, Governments developed and implemented Economic and Social Resilience Plans (e.g. In Senegal the FORCE COVID-19 amounted 1000 billion CFA francs, or 7% of the GDP.

### Positive consequences

#### Positive economic consequences

Most of the NPIs implemented in the four countries had economic consequences, some of which were positive and others negative. The manifestations of these consequences varied from one country to another depending on the specific context of the country, the level of implementation of these interventions and the measures taken locally to mitigate them. Table 1 below shows the positive economic consequences of the NPIs.

**Table 1:**
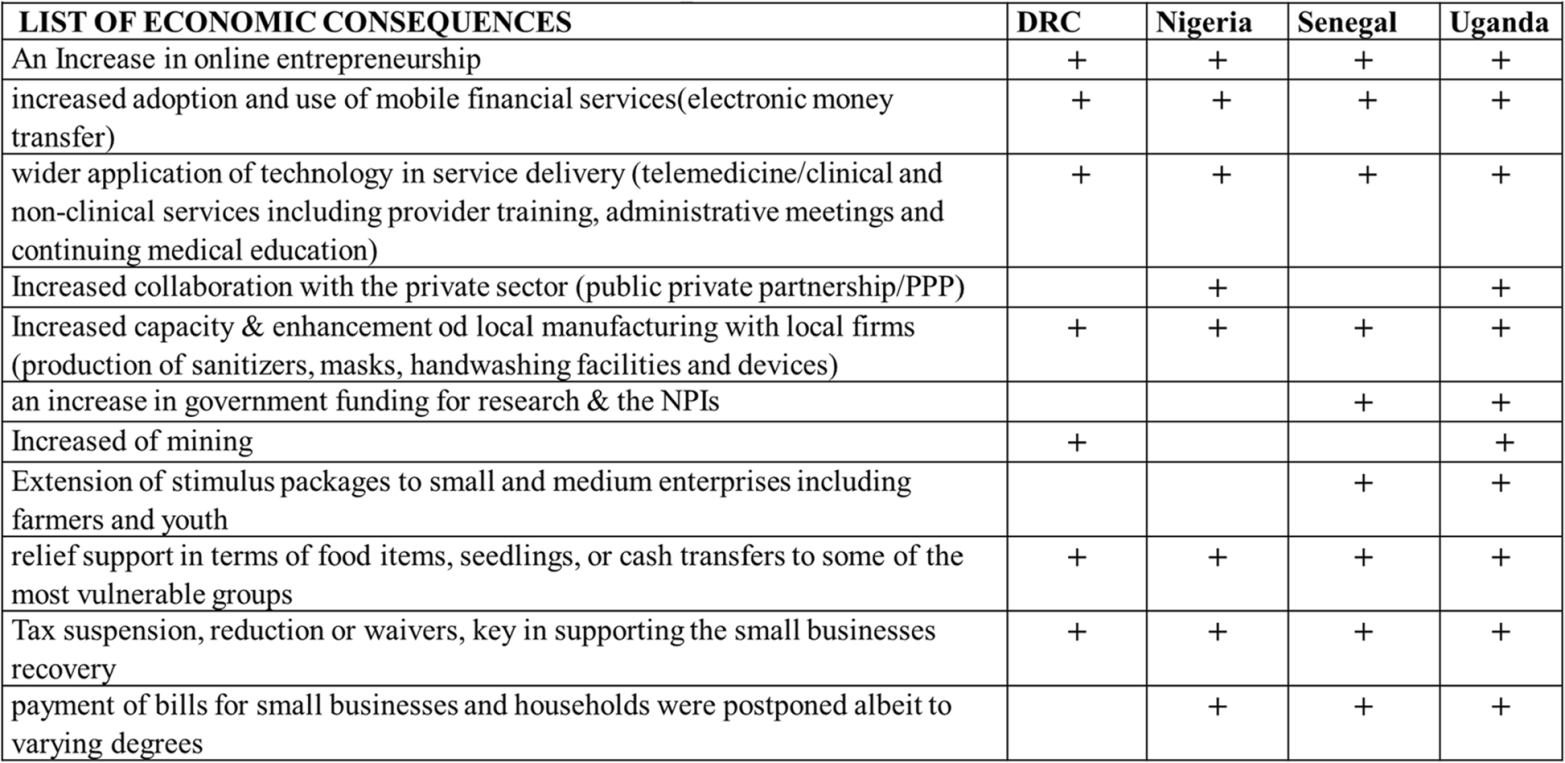
Positive economic consequences

Among the positive unintended effects was increased adoption and use of new communication technologies.

“*Of course, technology for meetings was more embraced as there was a ban on physical meetings. We also embraced technology for follow up of cases for instance in contact tracing. So, we saw a lot of innovations, which was a positive thing*.” (Policy maker, Ministry of Health in Uganda).

Thus, the use of mobile financial services and wider application of technology in service delivery to reduce the risk of spreading the virus, including the expansion of telemedicine, was observed to various degrees across the four countries. Even though telemedicine does not aim to replace a physical medical examination, it helped to reduce the exposure to and spread of the disease in the health facility. In DRC, the Project piloted telemedicine in Kingandu district [12] before its use during the pandemic. The use of electronic payment devices minimized physical exchange of goods and services including cash.

The KIs in DRC also noted that the pandemic had accentuated the use of electronic money payments. Similarly, several companies in Uganda adapted and innovated using online and digital systems, including door-to-door delivery of foods and medicines [13]. Key informants also noted that technology was adopted to facilitate surveillance and contact tracing activities and led to reduced costs.

Others turned to local solutions or practicable measures or repurposed their work to maintain their livelihood. For example, all four countries drew local resources via repurposing of industries to produce personal protective equipment, sanitizers and other products which were key in the COVID-19 response. Furthermore, the investment in research and innovations in providing low-cost solutions to intervention response was also key as well as the reinforcement of solidarity among community members.

Nigeria and Uganda reported another important positive economic effect of the NPI, which was the increased capacity and improved local manufacturing by local businesses to produce disinfectants, masks, and handwashing facilities, among others. Mobile Telephone Network (MTN) Nigeria, one of Nigeria’s large telecoms service providers unaudited financial results for the first quarter of 2020, showed increased data revenue by 32.4%. In Uganda and Senegal, public funding for research increased. These effects however varied across countries depending on the nature and extent of the NPIs as well as the fidelity with which they were implemented [14, 15].

#### Positive psychosocial consequences

The positive psychosocial consequences (Table 2) were:—increased community support and resilience (as communities came together to support each other). Also, hygiene practices improved through health promotion measures that were encouraged for pandemic response.

*“During this period, the most affected family members were assisted with some foods and money by others, meaning there was an intensification of solidarity. Others developed home gardens, and others started to sell masks or disinfectants as a means of livelihood” (*Key Informant Interview (KII), Adolescent Health Program in DRC).

**Table 2:**
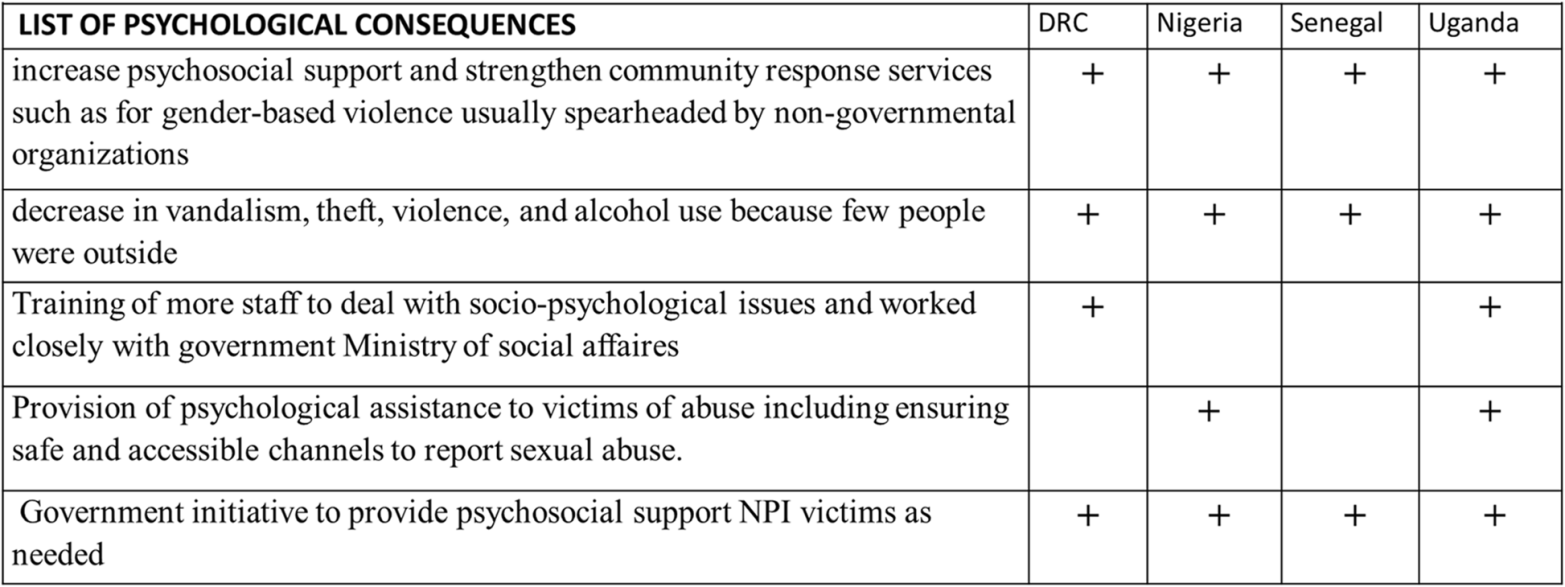
Positive psychosocial consequences

COVID-19 restrictions on movement led to family members spending more time together and bonding. Families and communities built local resilient capacity and coping mechanisms which contributed to their survival and mental health.

*“Due to my work, before I had just two hours to spend with my children, and with the containment measures, I spent more time with them, and this allowed me to teach them some manual work such as growing a small garden at home, and for girls, to cook food…”* (KI, Administrator of a private primary school in DRC).

The DRC, Nigeria, and Uganda also recorded a notable reduction in crime. In Uganda, the number of deaths from road accidents and attacks by thugs reduced significantly [16]. Furthermore, most of the KIs in DRC reported a decrease in vandalism, theft, violence, and alcohol use because few people were outside mainly due to the lockdown, closure of bars, night-club, and curfew and reinforcement of police patrols.

*“Some bars and restaurants were not complying with the closure measures. They were selling beer secretly. Consequently, the political authorities reinforced the police patrols, so that, the owners and even people who were caught in the outlets were forced to pay a fine. We observed a decrease in alcohol consumption and even of violence under the influence of alcohol.”* (KI, Military Health Zone of Kokolo Barrack in DRC).

A marked improvement in hygiene practices was reported in all four countries. Some KIs noted in DRC and Nigeria that the COVID-19 containment measures led to improvement of hygiene practices associated with disease prevention. They cited handwashing and improved cough etiquette to prevent the disease. These behaviors reduced the incidence of water and sanitation diseases. Across all the countries, there was increased physical activity such as brisk walking, jogging, cycling, and sporting activities among others in the neighborhoods as well as improved social interaction among community members during the lockdown.

#### Positive environmental consequences

Regarding the positive environmental consequences (Table 3), some interventions such as the lockdowns minimized air pollution while the personal protective measures improved hygiene practices [4]. Air quality improved in most countries, with Uganda recording improvements in water quality and a reduction in noise pollution. One study conducted in Kampala reported an improvement in air quality of up to 40% during the lockdown period [17]. In Nigeria, it was reported that due to lockdowns the levels of Nitrogen oxide(NO_2_) and Sulphur Dioxide (SO_2_) decreased by 1.1% and 215.5%, respectively while the levels of Ozone (O_3)_ increased [18]. In a survey conducted in Kaduna state, 86% agreed that the COVID-19 lockdown reduced greenhouse gas emissions (NO_2_) and improved air quality. The reduction in air pollution was also verified by the key informants.

“……*of course, with less cars on the road, we saw the least pollution, we saw more people going into Agriculture, people went back to the villages and started doing agriculture, even the water, I can imagine that we saw less sewage going into water sources and so on.*” (Policy Maker 1, Ministry of Health, in Uganda)

**Table 3:**
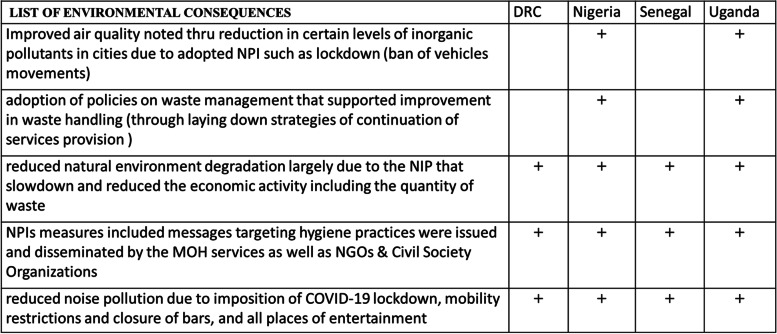
Positive environmental consequences

### Negative consequences

#### Negative economic consequences

The negative economic consequences (Table 4) were exacerbated by the existing precarious economic situation in all four countries prior to the pandemic. In developing countries in general, the informal sector is the most labor-intensive activity, particularly among adolescents and young adults. For example, in Sub-Saharan Africa (SSA), more than 80 percent of workers find their livelihoods in the informal sector as artisans and shop owners, fishers and divers, tailors and weavers, truck drivers and market sellers, among other informal jobs [19]. In all four countries, the economy is also heavily dependent on cross border activities and the sharp slowdown in domestic travel restrictions led to a sharp reduction in economic activity, with the tourism, transportation, construction, and retail sectors particularly affected. As shown in Table 4, the reduction of economic activities had negative economic effects. This was related to the slowdown of the economy in all the countries due to depressed global economy, border closures and movement restrictions and reduced exports. As a result, the economies did not grow as had been expected before COVID-19. Further, the slowdown of the economies negatively impacted businesses, increased unemployment rates as workers were laid off, reduced household income, and increased poverty levels. In DRC for instance, several factors contributed to the slowdown of the economy including:—COVID-19 restrictions limited exports, with a fall of copper and cobalt mineral prices due to border closures. Additionally, the remittances sent by African in Diaspora decreased significantly. The remittances from the Congolese in diaspora, who contribute about 1.5 billion USD annually to the DRC, was reduced. Thus, the DRC experienced negative growth in 2020 (-2.8%). Most of the key informants acknowledged that the closure of borders and suspension of international flights which hampered the commercial exchanges between DRC, USA, European and Asian countries negatively impacted the economy and increased poverty [20].

“*Impact of the restriction measures such as lockdown of Kinshasa (the capital city) as taken by the government were more on the economic sector and decreased productivity in general; there was no more wealth creation, and poverty was accentuated throughout the country*” (KI, Ministry of Gender, Family, and Children, DRC).

**Table 4:**
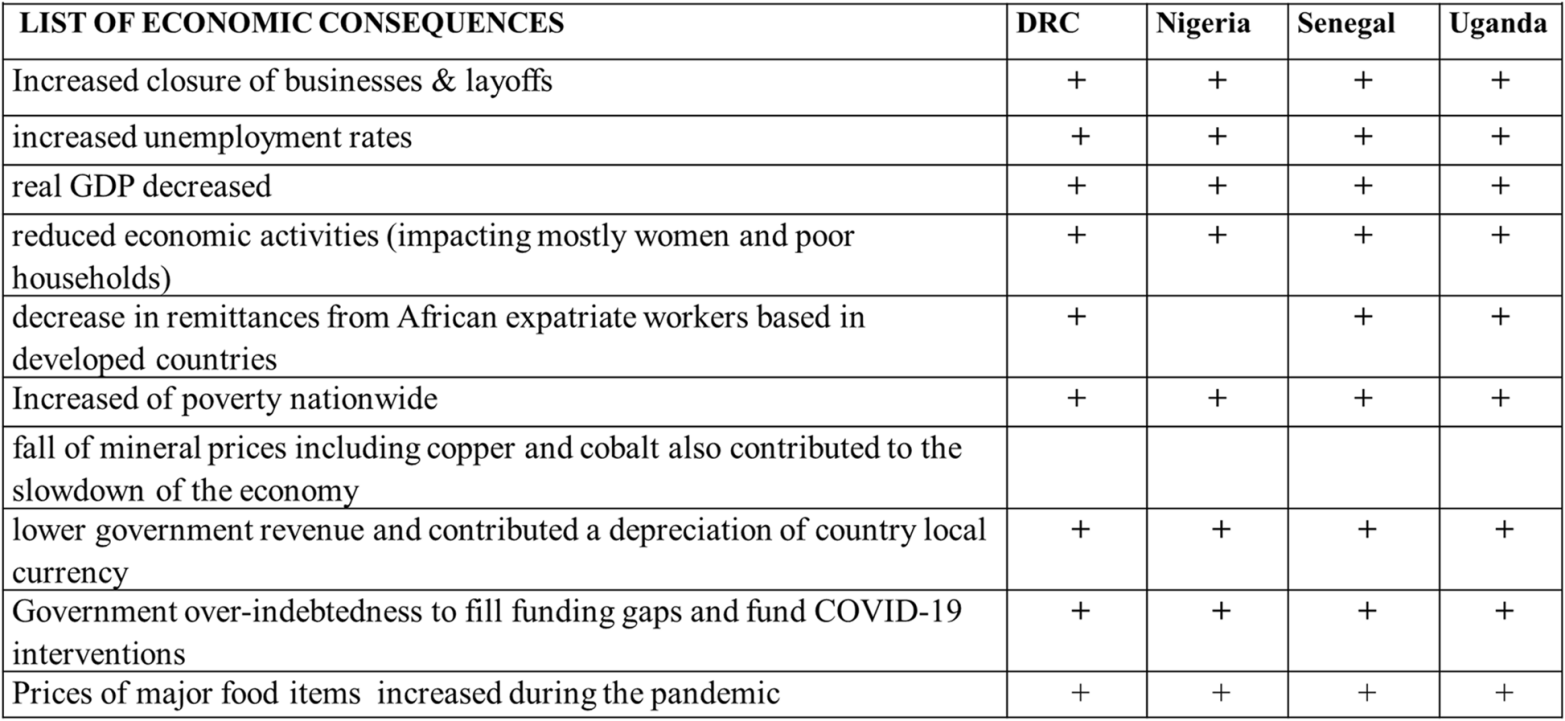
Negative economic consequences

During the lockdown business activities were disrupted, increasing unemployment as companies were asked to reduce the number of workers in order to maintain physical distancing [21]. A survey conducted in July 2020 in DRC found that 94% of firms reported that their revenues had dropped compared to the pre-pandemic levels, and over 95% said their businesses declined wholly or partially. As cases dropped in the second half of 2020 and lockdown and movement measures were lifted, the business environment also appeared to improve. In Nigeria, Andam and his colleagues found that the total Gross Domestic Product (GDP) and Agri-food system GDP dropped by 23% and 11% during the lockdown respectively. Household incomes reduced by a quarter, leading to a 9% point increase in the national poverty rate by the end of 2020 (Andam, Edeh et al. 2020) [22]. Senegal’s real GDP growth was reported to have increased by only 0.9% in 2020, compared with 5.3% a year earlier, with unemployment and a lack of economic opportunities [23]. Its GDP growth was projected at 1.1% for the year 2020, while it was expected at 6.8%. In Uganda, the country real GDP grew at 2.9% in FY2020, less than half the 6.8% recorded in 2019 financial year, mainly attributed to the effects of the COVID-19 pandemic [24].

To reverse the negative economic effects, governments borrowed more to fill funding gaps and fund COVID-19 interventions and suspended, reduced, or waived taxes. In Senegal and Uganda, additional support was provided to businesses. The International Monetary Fund (IMF), the World Bank, other multilateral organizations and bilateral partnerships provided variable financial support to each of the four countries to support the implementation of the NPIs, strengthen macroeconomic stability, address governance and pervasive poverty, while trying to mitigate the unintended consequences of the control measures.

The socio-economic consequences were mostly negative in all four countries. In Uganda, approximately 100,000 jobs were lost in the formal sector and 4.4 million in the informal sector especially in the north and the southwest of the country. The biggest loss was in Kampala, the capital [25]. With the enforcement of social distancing, cross border trade in DRC stopped [26]. People living on informal subsistence activities (market, gardening, small trade, street vendors, etc.) in rural and urban areas were majorly affected by the restrictive measures. Small businesses reoriented their sales and began to sell masks and disinfectants to augment their income. This phenomenon was mentioned by interviewees as reflected by quotation recorded from the KIs interview in DRC.

*“Lockdown and other related restriction measures such as suspension of travels between Kinshasa and other provinces, closure of restaurants, bars, night-clubs led to a significant decline in household income. This accentuated the precarious financial situation of most of the families in the informal sector”* (KI, Ministry of Gender, Family, and Children, DRC).

The difficult socio-economic issues were worse in households headed by women. In DRC for example, 15% of women lost their jobs compared to 9% of men. The reasons for the loss of employment were: confinement (71.0%), cessation of activity (27.3%), reduction of staff (10.3%) and illness (6.0%). In August 2020, an ÉLAN RDC study found that two-thirds of 2,200 households surveyed had experienced a reduction in their monthly income compared to the period before the pandemic [27]. The KIs acknowledged that the COVID-19 restrictions led to scarcity of essential foods and basic commodities. Prices increased in DRC and Senegal that imported most of the food their populations consume including rice, maize flour, sugar, frozen fish and meat.

*“The restriction measures limiting movement led to scarcity of food and ultimately to the rising of prices; the majority of the population was then unable to buy foods during this period; getting enough to eat was the primary concern and it was most of the time impossible”* (KI, Adolescent Health Program in DRC).

Support measures given to low-income households included: -food aid which was distributed to the most vulnerable in the urban areas of Uganda. In Nigeria and Uganda, in addition to food distribution, cash transfers were provided to households. In the DRC, agricultural seeds were distributed, and other support was provided mainly to displaced people, host communities and refugees, while in Senegal, in addition to this aid, there was a ban on raising commodity prices.

#### Negative psychosocial consequences

Several psychological and behavioral consequences (Table 5) including early marriages, sexual violence against young girls, domestic violence, increased pregnancies and gender-based violence, among others, were reported in all four countries. Since the onset of the COVID-19 pandemic and implementation of mitigation measures in DRC, studies reported an increase in cases of children who had been forced into marriage, arbitrarily detained, harassed, pillaged, forcibly enlisted, or exposed to sexual violence. There was also increased cases of sexual violence against young girls in the Eastern provinces of the country (North Kivu, South Kivu and Ituri) where armed conflicts and insecurity were prevailing and children forcefully recruited into armed groups [28].

*“A few months after we declared the reopening of the institutions and others, we noticed that many girls were pregnant and others had carried out abortions. We also heard people telling us that such and such a girl was pregnant and was trying to have an abortion. These are stories that we experienced and heard”* (KII, student in DRC).

**Table 5:**
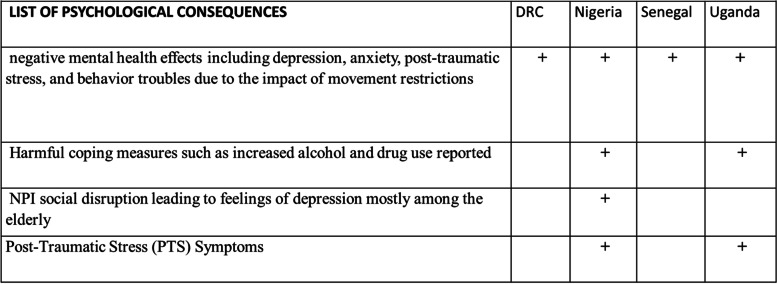
Negative psychosocial consequences

The movement restrictions led to an increase in sexual and gender-based violence, teenage pregnancies, and early marriages, which mainly affected girls. The economic crisis increased the exposure of girls to sexual relations, to having multiple sexual partners to meet their basic needs. In Nigeria, in the first two weeks of lockdown, intimate partner violence cases rose from 346 in March to 794, depicting a 56% increase in just two weeks of lockdown. Some of these incidents of violence have tragically resulted in the death of victims [29]. Findings from key informant interview corroborated this. One respondent said.

*“There was increase in the number of rape cases, armed robbery cases, gender-based violence. …...”* (KII-18, Nigeria)*.*

During the lockdown, men in Uganda were reportedly victims of emotional abuse by their partners due to inability to provide for their families [30]. Anxiety disorders also escalated due to movement restrictions, inability to access counseling or social support services by the victims and the inability to escape abusive partners [31]. Key informants stated that the lockdown led to loss of jobs, high drug abuse, and failure to adopt to the new norms, which resulted in mental health problems.

“*We had reports of increased mental disturbances amongst individuals affected variously either through not being given the chance to earn a living for their families or some took to substance abuse as a way of coping, so the mental disturbances suddenly came up”* (Policy maker 3, Ministry of Health, in Uganda).

Another consequence was the inability of the survivors of GBV to access affordable quality services, mainly due to the limited availability of GBV referral centers (Peterman, O’Donnell et al. 2020) [32]. In Uganda, there were reports of increased teenage pregnancy especially in the Eastern and Northern districts. It was estimated that over 4,000 teenage pregnancies were registered in Northern Uganda during the first 5 months of the COVID-19 lockdown [33].

“*… teenage pregnancy escalated as people stayed home longer, children in the community not schooling and many were abused. This morning I have just been reading about 17,000 pregnancies in Gulu region alone that have occurred amongst teenagers”* (Policy maker 3 Ministry of Health, in Uganda).

In Uganda, alcohol-related risk behaviors such as partner violence increased. A study that involved a total of 556 Ugandan women aged 13–79 years (mean age of 33.4 years) during the COVID 19 lockdown, concluded that lockdown measures in Uganda may have mitigated increased alcohol consumption. Intimate Partner Violence (IPV) as generalized self-efficacy increased (adjusted OR = 0.95; 95% CI = 0.91– 0.99; *p* = 0.0308). It was associated with four times greater odds of recent physical intimate partner violence (IPV) (a OR 4.06, 95% CI = 1.65–10.02, *p* = 0.0024), while participants who had not experienced any IPV in the past six months had higher perceived self-efficacy than those who did report recent IPV (z = -2.47, *p* = 0.0133) [34]. In a survey conducted in Senegal, 72% of respondents said that their psychological/mental/emotional health (stress, anxiety, etc.) had been affected by the COVID-19 pandemic. Women (76%) were much more likely to report being emotionally and psychologically affected by COVID-19 than men (68%) [35]. In a cross-country study on the impact of the lockdown measures on mental health in 67 countries including Nigeria, the lockdown measures accounted for about 4.4% of the increase in depression within the population sampled. Personal health beliefs about the lockdown negatively influenced the mental health status of the people while general health beliefs positively influence it [36].

In DRC, Médecins du Monde reported a doubling of the number of cases of sexual gender-based violence (SGBV) received in its health centers in Kinshasa between April and June, 2020 [37]. According to KIs interviewed, the inability of husbands to cope with the stress caused by professional or precarious financial situation (reduced income) that limited their capacity to meet basic household needs contributed to the high violence rate against women observed.

*” What we have observed is that there was more sexual and physical violence against women perpetrated by their husbands. You know it is not easy to deal with stress caused by lack of money, and if every time your spouse is requesting for money while you do not have it, you may react more violently.”* (KII, Civil Society Organization in DRC).

The NPIs also contributed to an increase in anxiety and fear [38]. Psychosocial effects have been observed in DRC during this pandemic including an increase in anxiety and fear following COVID-19 measures taken by the government [39]. An online survey conducted among 342 participants from March to May 2020 reported the prevalence of depressive symptoms in DRC at 27.02% [40]. This high prevalence of depressive symptoms was attributed to the degradation of perceived health between the pre-pandemic and COVID-19 period, increase in symptoms of anxiety especially among boys, stress resulting from social isolation or anxiety in waiting for test results. The factors associated with experience of anxiety and depression were old age, female gender, lack of occupation, and isolation [41]. In Nigeria, instead of the reduction of crimes as observed in the other countries, there was an increase in social ills, unrest, and theft due partly to frustration resulting from loss of jobs, and socio-economic difficulties due to the NPIs [42].

Confinement measures, job losses and restrictions on movement were cited, in a recent DRC paper, redundancy and idleness at home was one of the main explanations for the increase in pregnancies, as men and women spent more time together at home, leading to an increase in sexual activity [43].

Measures have been taken to increase psychosocial support and strengthen community-based intervention services such as gender-based violence prevention, usually led by non-governmental organizations.

#### Negatives environmental consequences

The negative environmental consequences (Table 6) of the NPIs were mostly related to waste generation and poor disposal that increased in all areas, and attributed to movement restrictions, COVID-19-related waste generation, and the diversion of funds for sanitation services in some countries. In DRC and Uganda, policies on waste management supported improvement in waste handling through laying down strategies of continuation of services provision following increased COVID-19 waste and their poor disposal. In Nigeria, the solid waste from COVID-19 resources such as face mask, gloves, empty hand sanitizer containers and other plastic personal protective gears constituted a national embarrassment even though the country had only recently approved a national solid waste management policy. In Uganda, hazardous waste was generated due to the establishment of quarantine centers and isolation facilities to manage COVID-19 suspects and patients, but their disposal was a challenge.

**Table 6:**
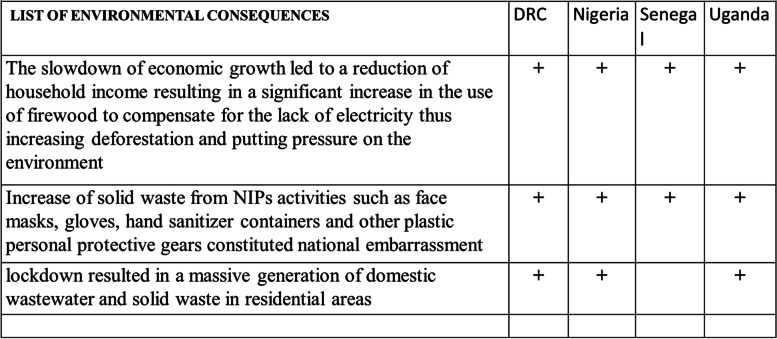
Negative environmental consequences

## Discussions

The unintended consequences of NPIs implemented in the four countries: curfews, border closures, physical distancing, bans on gatherings, wearing masks, systematic and frequent hand washing, have been positive and negative in the socio-economic, psychological, and environmental spheres. The positive unintended effects included reduction in crime rates and road accidents, particularly in the DRC, Nigeria and Uganda, as well as a reduction in air pollution, which Uganda also reported. The implementation of some NPIs, particularly the curfew, led to enhanced community cohesion to support each other, demonstrated increased resilience. The whole world had adopted curfews or lockdowns to limit the transmission of the coronavirus by restricting human mobility and commercial activities. This sudden halt to all types of social, economic, industrial and urbanization activities has led to improved air quality, cleaner rivers, less noise, calm and undisturbed wildlife [44].

The positive environmental consequences of the NPIs found in our study, including improved air, water, physical environmental and noise pollution quality, have been reported in other studies [45]. Air quality has improved in most countries, with Uganda recording further improvements in water quality and quantity and a reduction in noise pollution. This positive effect on the environment has been reported throughout the world as the first positive aspect of COVID-19 restrictive measures [45, 46]*.* China recorded an 85% increase in days with good air quality in 337 cities between January and March 2020. Better hygiene and potential effects on improved health has also been reported [47].

The increased community support and resilience as communities came together to support each other in all four countries, has been reported elsewhere. The self-isolation and related challenges created the resurgence of the desire for relationships and socialization. People found new ways to meet this need for interconnection. In Italy, for example one of the countries that was most affected, people united their instruments and voices to create music from their balconies [48]. In addition, people used social media platforms such as the Facebook group and others to connect [45].

With regard to the socio-economic consequences of the NPIs, they were mostly negative, in the sense that they mainly led to the slowdown of the economic sector in general and in particular the informal sector. The most worrying consequences were those that were highlighted in all countries and that necessitated measures to reduce their effects on most vulnerable populations. These negative consequences affected countries at varying levels and degrees except for the socio-economic effects, which were apparently similar in all four countries. There was a reduction in household income orchestrated by job losses or a reduction in economic activities that had the greatest impact on women and the poorest households. The slowdown in the informal sector, which employs most women and low-income households, was notable and further impacted on their incomes and the well-being of families. Many of these households also faced increased food insecurity. There was also critical psychosocial consequences such as an increase in sexual and gender-based violence, teenage pregnancies, early marriages, an increase in poor mental health conditions, as well as an increase in waste generation with poor disposal, among others. These negative consequences such as the slowdown in the economy, the fall in GDP, increased unemployment, found in the four countries, have been reported elsewhere across the world and especially in Low and Middle Income Countries (LMIC) [46, 49]. In the African countries south of the Sahara, apart from South Africa, although less affected from an epidemiological point of view, the pandemic has had serious repercussions on the economic and social sectors in almost all countries. It threatens to push up to 58 million people into extreme poverty. This setback, according to Kohnert, Dirk will be felt for decades to come [50].

Whereas countries implemented various mitigation measures to minimize the socioeconomic stress on the most vulnerable groups, these were not sustained and there did not seem to be a very detailed criteria for their selection and a database or other mechanisms to easily reach them in an equitable manner.

The psychosocial consequences of the NPIs studied in the four countries are among the major concerns of these unintended consequences. Although on a positive note intervention led to the strengthening of community resilience, they caused serious psychosocial disruptions such as alcoholism, depression, anxiety, sexual violence, unwanted teenage pregnancies. They were linked to measures to restrict movement and freedoms. Brooks et al. revealed in their work on the subject, that separation from loved ones, loss of freedom, uncertainty about the status of the disease and boredom can, on occasion, create dramatic psychosocial effects [51]. The duration of confinement itself is a stressor and longer duration may lead to post-traumatic symptoms, avoidance behaviors and anger [52].

The restrictive measures affected consumer products, particularly pharmaceutical products. They restricted consumers spending across all industries including social services, leisure, and hospitality, among others. Further, consumers are less inclined to spend more, with many expecting their household income to continue to fall in the coming months [53–55]. Some pharmaceutical products can be harmful to the environment especially if they are not managed appropriately or if their date of consumption has expired [56]. An Organization for Economic Co-operation and Development (OECD) report also points out that conventional purification systems are not designed to eliminate pharmaceuticals. As their volumes and varieties increase, the risk of finding more and more residues in the water also increases [57].

“The economic consequences of this pandemic are very different depending on the countries, partly for reasons directly linked to Covid-19 and the way it has affected them. The countries most affected are also those that will experience the most severe economic downturns. It is thus Asia that is doing the best, as well as part of Africa, even succeeding for certain countries such as China, Vietnam, Kenya, or the Cote d’Ivoire in achieving growth in their GDP according to the latest forecasts from the International Monetary Fund (IMF)” [58]. The governments should develop reliable systems, accurate measures of vulnerability and effective monitoring procedures to ensure equitable socio-economic support and mitigation during epidemics. The lack of systematic evaluation of the unintended consequences of the NPIs and mitigation measures has been pointed out by several authors [49].

### Lessons learned on NPIs unintended consequences

Based on our work, the following are some of the key takeaways we ought to consider for the future:

These lessons drawn from our study include the following.

- *Select NPIs having in mind potential unintended consequences:* Owing to the magnitude and importance of unintended consequences, due consideration should be given to the likely consequences of all NPIs, and measures taken to mitigate these consequences. The unintended consequences of COVID-19 were gendered with females and households of low socioeconomic status and female-headed most impacted. Future epidemic response should consider gender as a key dimension in selecting interventions.
- *Need for Multisectoral approach:* Given the multisectoral impact of COVID-19 and how the unintended consequences of NPIs cut across all sectors, there is a need for multisectoral action in epidemic response including the planning and implementation of NPIs and mitigation measures.
- *Relief Support to the Vulnerable:* Several vulnerable groups in all four countries were offered relief support in terms of food items, seedlings, or cash transfers. This support enabled households and communities to cope with the effects of the epidemic.
- *Build Community Resilience:* Most communities demonstrated resilience and worked together to support each other to deal with the pandemic and its effects. Social support in the form of family and community closeness was essential and efforts should be geared towards strengthening coping mechanisms and resilience.
- *Strengthen data systems:* A key part of the response in these countries was to extend social support to vulnerable groups but governments struggled to obtain a robust and accurate database of those that needed to be prioritized. Countries ought to build efficient systems to identify and equitably support vulnerable groups.
- *Psychosocial Support:* The pandemic and its response measures exacerbated mental health problems including depression, anxiety, and suicides among others. Psychosocial support should be at the forefront of all epidemic response and include the use of technology.
- *Draw on Local Resources:* The COVID-19 response was characterized with countries looking inwardly for solutions as many countries shut their borders and focused on their response, and this affected cross border movements and trade. Future responses should identify local resources which could be utilized to cost-effectively produce materials but also integrate a broader cross border coordinated continentwide response.
- *Research, Innovations, and Technological Solutions:* Technology adoption and use were very key in the response to COVID-19 and some of the interventions implemented to alleviate its negative effects. Long-term and sustained investments in research and innovations and use of technology in health, education, and other sectors is needed as part of a comprehensive preparedness and response plan in Africa.
- *Government Economic measures and Tax exemptions:* Several governments took measures to suspend, subsidize or exempt taxes, especially for small businesses and this was noted to be key in supporting their recovery. Such interventions are important to community livelihoods.
- *Ensure the Continuity of Essential Services:* In Nigeria, there was a neglect of provision of sanitation and waste management services with funding support redirected which negatively impacted environmental aesthetics. Future responses should ensure that the continuity of such essential services is planned for in advance and their continuity guaranteed.
- *Lifting of Restrictions Requires Careful Planning:* Although some positive unintended consequences were registered from the NPIs, some of these were lost immediately after they were lifted. Careful planning and adequate countermeasures are required to sustain the positive gains from the implementation of the NPIs.

## Conclusions

The NPIs implemented for COVID-19 prevention and control had both positive and predominantly negative unintended consequences which cut across economic, socio-economic, psychosocial, and environmental spheres. The positive consequences included increased adoption and use of mobile financial services and wider application of technology in services, increased community support and resilience and reduced crime rate. On the contrary, the implementation of NPIs contributed to a slowdown of the economy, loss of employment, closure of businesses, and increased poverty levels. Moreover, household incomes were negatively and severely impacted and food insecurity increased. An increase in mental health conditions was also registered. Whereas the NPIs were instituted to protect populations from disease, the resultant socioeconomic disruptions affected critical determinants of health and could result in major health consequences and poor health outcomes in the medium to long term. Efforts are urgently needed to effectively address the negative effects of the NPIs and their resultant consequences early enough to reverse their subsequent negative effects.

In the future, the criteria for choosing these NPIs interventions should consider their possible unintended consequences and mitigation measures for the negative ones. Additional protection should especially be afforded to those that are most vulnerable to these consequences, building community resilience as well as providing psychosocial support. More experts should be trained, and more resources allocated for mental health services provision and the application of technological solutions. The use of technologies such as online or telephone assistance should be considered in accordance with the control measures of COVDI-19. In the same way the laws against violence and the necessary support should also be strengthened and enforced to combat gender-based, sexual, and physical violence and deter perpetrators. Governments and local authorities must finally ensure the continuity of essential services, including regular waste collection and disposal and proper planning for the management of COVID-19-related waste, including masks, face shields and medical waste.

## Data Availability

The datasets during and/or analyzed during the current study available from the corresponding author on reasonable request.

## Abbreviations

COVID 19: Coronavirus Sars-Cov-2
DRC: Democratic Republic of Congo
USA: United States of America
NPI: Non-Pharmaceutical Intervention
RNA: Ribonucleic acid
CFR: Case Fertility Rate
WNTRA: A Worldwide Non-pharmaceutical Interventions Tracker for COVID-19 software
CFA: « Communauté Financière Africaine» *(in the countries of the West African Economic and Monetary Union and the African Financial Cooperation franc in the countries of the Central African Monetary Union)*
GDP: Gross domestic product
KI: Key Informant
MTN: Mobile Telephone Network
MOH: Ministry of Health
NGO: Non-governmental Organizations
NO_2_: Nitrogen Sulfate
SO_2_: Sulfur dioxide
SSA: Sub-Saharan Africa
USD: United States dollar
IMF: International Monetary Fund
IPV: Intimate Partner Violence
CI: Coefficient Interval
LMIC: Low- and middle-income countries
OECD: Organization for economic co-operation and development
